# Selection of Reliable Reference Genes for Gene Expression Normalization in *Sagittaria trifolia*

**DOI:** 10.3390/genes14071321

**Published:** 2023-06-23

**Authors:** Jing Tang, Enjiao Li, Jiexia Liu, Zhiping Zhang, Bing Hua, Jiezeng Jiang, Minmin Miao

**Affiliations:** 1 College of Horticulture and Landscape, Yangzhou University, Yangzhou 225009, China; 2 Joint International Research Laboratory of Agriculture and Agri-Product Safety of Ministry of Education of China, Yangzhou University, Yangzhou 225009, China; 3 Key Laboratory of Plant Functional Genomics of the Ministry of Education/Jiangsu Key Laboratory of Crop Genomics and Molecular Breeding, Yangzhou University, Yangzhou 225009, China

**Keywords:** reference gene, RT-qPCR, expression stability, arrowhead, different tissue, corm development

## Abstract

Real-time quantitative PCR (RT-qPCR) is a method with high sensitivity and convenience that has been extensively used to analyze the expression level of target genes. A reference gene with a highly stable expression is required to ensure the accuracy of experimental results. However, the report on appropriate reference genes in arrowheads (*Sagittaria trifolia*) is still limited. In this study, eight candidate reference genes (*ACT5*, *UBQ*, *GAPDH*, *CYP*, *NAC*, *IDH*, *SLEEPER* and *PLA*) were selected. The candidate genes were employed in a RT-qPCR assay in different tissues at different developmental stages of the same tissue (including corm, leaf and leafstalk) in arrowheads. Five statistical algorithms, GeNorm, NormFinder, BestKeeper, delta cycle threshold (ΔCt) and RefFinder, were used to evaluate the stability of these genes’ expressions in order to identify the appropriate reference genes. The results showed that *UBQ* was the optimum reference gene in leaf, leafstalk, root, stolon and corm, *IDH* exhibited the most stable expression during the expansion of corm, *UBQ* and *PLA* were the most stable reference genes in developmental stages of leaf and leafstalk, respectively. Finally, the reliability of reference genes was further confirmed by the normalization of *PDS* and *EXP1* genes under different arrowhead tissues and developmental stages of corm, respectively. This study constitutes important guidance for the selection of reliable reference genes for analyzing the tissue- and developmental-stage-specific expression of genes in arrowheads.

## 1. Introduction

Real-time quantitative PCR (RT-qPCR) is an approach that uses fluorescence signals to monitor the total amount of products after each polymerase chain reaction (PCR) cycle [1]. Due to its high accuracy and strong specificity, it has been widely applied to analyze gene expression levels [2,3]. The gene expression analysis by RT-qPCR is affected by many factors, such as RNA quality, cDNA synthesis efficiency, primer specificity, and the stability of reference genes [4,5]. The expression level of the target gene can be calibrated by using stably expressed reference genes as standard, so as to reduce the difference between the target gene expression and the true value [6]. The stability of reference genes exerts a decisive effect; unstable reference genes will lead to erroneous results in gene expression quantification [7]. Selecting appropriate reference genes to standardize the expression of target genes is required to ensure the accuracy and reliability of RT-qPCR results [8,9].

Reference genes are a class of genes that is stably expressed in various tissues and cells, or stable in the same tissues and cells at all stages of plant growth. The expression of these reference genes will not be affected by the variation of external environmental factors and experimental conditions [10,11]. The commonly used reference genes include actin gene (*ACT*), glyceraldehyde-3-phosphate dehydrogenase gene (*GAPDH*), tubulin gene (*TUB*), ubiquitin gene (*UBQ*), ribosomal RNA encoding gene (*18S* rRNA) and others [12,13]. Reference genes differ between species, and different experimental conditions also require different reference genes. Therefore, the study on the stability of reference gene expression in the materials from different groups is prerequisite and crucial for gene expression normalization.

Arrowhead (*S. trifolia* L.) is an herbaceous aquatic plant of the genus *Sagittaria* in the Alismaceae family. Arrowhead contains arrow-shaped leaves, white flowers, and expanded corms from an underground part. The corms show ovoid or spherical shape, and are the edible organ of arrowheads with high nutritional and medicinal values [14]. It is reported that arrowhead contains a variety of colchicine, thus showing several effects, including clearing heat, reducing swelling, preventing cancer and fighting cancer [15]. The nutrients in arrowhead corms are mainly composed of starch, protein, fat, carbohydrates and fiber [16]. Cultivated arrowheads are mainly distributed in the middle and lower reaches of the Yangtze River, South China and Southwest China, and are a kind of characteristic vegetable favored by consumers due to their abundant nutrition. They are also of great value in enriching the variety of vegetables on the table.

For arrowheads, the research on their molecular biology is lagging far behind other species. The analysis of gene expression is the most fundamental and frequently used technical tool in molecular biology research. However, there is still no consensus on which genes can be applied as reference genes in RT-qPCR experiments of arrowheads. In this study, eight potential genes (*ACT5*, *UBQ*, *GAPDH*, *CYP*, *NAC*, *IDH*, *SLEEPER*, and *PLA*) were selected as candidate genes. Then, we tested these genes expression levels in different tissues and three developmental stages of the same tissue (corm, leaf and leafstalk), and then performed the normalization assay and verification to identify stable reference genes for future RT-qPCR analysis.

## 2. Materials and Methods

### 2.1. Plant Materials

**Figure 1:**
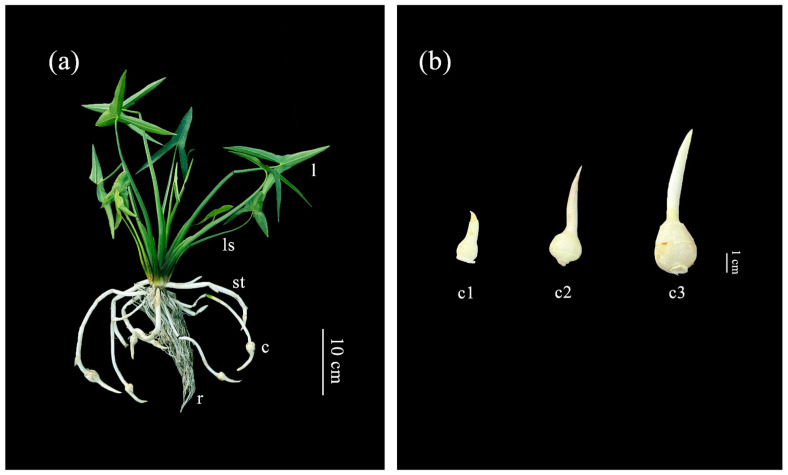
The definitions of tissue and corm developmental stages in arrowheads. (**a**) Five tissues of arrowheads. l: leaf, ls: leafstalk, st: stolon, r: root, c: corm. (**b**) Three stages of arrowheads corm development (c1–c3).

In this experiment, the *S. trifolia.* L. cv. ‘Baimati (BMT)’ was used as the material, which was planted in the aquatic vegetable experimental field of Yangzhou University. The arrowhead plants with 3–4 leaves were planted in the non-porous plant pot containing soil and maintained a 5 cm water layer on the surface of the soil. Early corm formation occurred at 6–7 weeks after planting. Here, the corm developmental stage was divided into three stages, including stage 1 (c1, 50 days after planting), stage 2 (c2, 70 days after planting) and stage 3 (c3, 90 days after planting). Leaf and leafstalk growth was divided into three stages, namely stage 1 (30 days after planting), stage 2 (50 days after planting) and stage 3 (70 days after planting). Different tissues (leaf, leafstalk, root, stolon, corm) were collected at stage 2 of corm development. The tissues, including corm, leaf and leafstalk, at three developmental stages were also harvested at three stages, respectively (Figure 1). Obtained samples were stored at −80 °C for subsequent experiment.

### 2.2. RNA Extraction and cDNA Synthesis

RNA was extracted using the Plant Total RNA Extraction Kit (Pudi, Shanghai, China). The concentration and quality of RNA was determined using a One-Drop^TM^ spectrophotometer (Thermo Fisher, Waltham, MA, USA). The OD_260/280_ of each RNA sample was 1.8~2.1. Purified RNA was stored in an ultra-low-temperature refrigerator at −80 °C. Then, 1000 ng RNA was used for reverse transcription to generate cDNA, and the obtained cDNA was stored at a −20 °C condition. All experimental procedures were conducted according to the manufacturer’s instructions. Three biological replicates were set for each sample.

### 2.3. Primer Design

The ORF (open reading frame) of eight reference genes (*ACT5*, *UBQ*, *GAPDH*, *CYP*, *NAC*, *IDH*, *SLEEPER*, and *PLA*) was retrieved from our transcriptome database and cloned from the ‘BMT’ cDNA. Specific primers of these genes were designed using Primer 6.0 software (Premier Biosoft, Palo Alto, CA, USA). The parameter for primer design was set as follows: melting temperatures (Tm) of 54–60 °C, GC content of 45~55%, primer length ranging between 18 and 25 bp, an amplicon length range of 100~250 bp. Designed primers for all target genes were synthesized by General Biology corporation (Chuzhou, Anhui, China). The sequences of all primers are listed in Table 1.

### 2.4. Primer’s Performance Analysis and RT-qPCR Test

The fragment of candidate reference genes was amplified using designed primers and a cDNA template from arrowhead corms. The amplification reaction system was 20 μL, including 10 μL of Taq PCR SuperMix (TransGen, Beijing, China), 1 μL of forward/reverse primers (10 μM), 2 μL of cDNA and 6 μL of ddH_2_O. The reaction conditions were as follows: pre-denaturation at 94 °C for 5 min, then denaturation at 94 °C for 30 s, annealing at 60 °C for 30 s, extension at 72 °C for 30 s, a total of 35 cycles, and finally 72 °C for 10 min. The amplified products were analyzed by 1% agarose gel electrophoresis to detect the uniqueness of the amplified bands. The amplification efficiency of primers was detected by a qPCR assay using a 10-fold diluted series (10×, 10^2^×, 10^3^×, 10^4^× and 10^5^×) of cDNA of arrowhead corms. The total reaction mixture for qPCR was 10 μL, including 4 μL of cDNA, 5 μL of iTap Universal SYBR Green Supermix (Bio-Rad, Hercules, CA, USA) and 0.5 μL of forward/reverse primer (10 μM). Reaction conditions involved pre-denaturation at 95 °C for 3 min, followed by 39 cycles at 95 °C/10 s and 60 °C/30 s. The amplicon was heated from 65 to 95 °C to obtain the dissociation curve. Three technical repetitions were set for each sample. The cDNA of all arrowhead tissues was used as the template for the analysis of the melting curve. The specificity of primer was further determined by melting curves. In addition, Cq values were used to draw standard curves, and the primer amplification efficiency (E) and correlation coefficient (R^2^) were analyzed. The computing formula of primer amplification efficiency was % E = (10^[−1/slope]^ − 1) × 100%. The calculation of amplification efficiency requires that the standard curve has a satisfactory linear relationship (R^2^ > 0.99), and the amplification efficiency of all primers meeting the requirements of qPCR detection should be between 90% and 110%.

The expression levels of candidate reference genes in five plant tissues and three developmental stages of the same tissue (corm, leaf and leafstalk) were performed by RT-qPCR analysis, using the CFX96 PCR system (Bio-Rad, Hercules, CA, USA). Three biological replicates, each with three technical replicates, were set for the samples.

### 2.5. Statistical Analysis of Gene Expression Stability

In order to select the reference genes with high expression stability, four statistical algorithms were employed. Firstly, the M value (gene expression stability measurement) was computed using GeNorm to screen stable reference genes [17]. NormFinder software was used to reanalyze and calculate the S value (Normfinder stability) [18]. After evaluation by SD (standard deviation) and CV (coefficient of variation) using BestKeeper, the reference genes were ranked [19,20], and then the SD value was analyzed again by ΔCt [21]. Finally, RefFinder [22] (available online: http://blooge.cn/RefFinder/, accessed on 15 April 2023) was used to synthetically analyze data from all algorithms. The reliable reference genes in different tissues and developmental stages were screened. All algorithms were used according to the manufacturer’s instructions.

### 2.6. Expression Analysis of PDS and EXP1 Genes

*PDS* (encoding phytoene desaturase) and *EXP1* (encoding one expansin) were chosen as target genes to verify the reliability of selected reference genes and their ORF sequences were listed in Figures S1 and S2. *PDS* was used to test the reliability of reference genes in different tissues, and *EXP1* was used to verify the reference gene for corms at three developmental stages. The expression levels of two target genes, *PDS* and *EXP1*, were normalized using the top two stable and least stable reference genes. The reaction conditions of RT-qPCR consulting in the procedure are described above. Relative expression levels of *PDS* and *EXP1* were calculated by the 2^−ΔΔCt^ method. Three biological repetitions and technical repetitions were set for each reaction. The primers for gene expression verification of *PDS* and *EXP1* are shown in Table 2.

### 2.7. Data Analysis

The significance of data was analyzed by one-way ANOVA using Duncan’s method. SPSS v20.0 software was used to conduct one-way ANOVA. Values of *p* < 0.05 were considered statistically significant.

## 3. Results

### 3.1. Specificity and Amplification Efficiency of Primers

**Figure 2:**
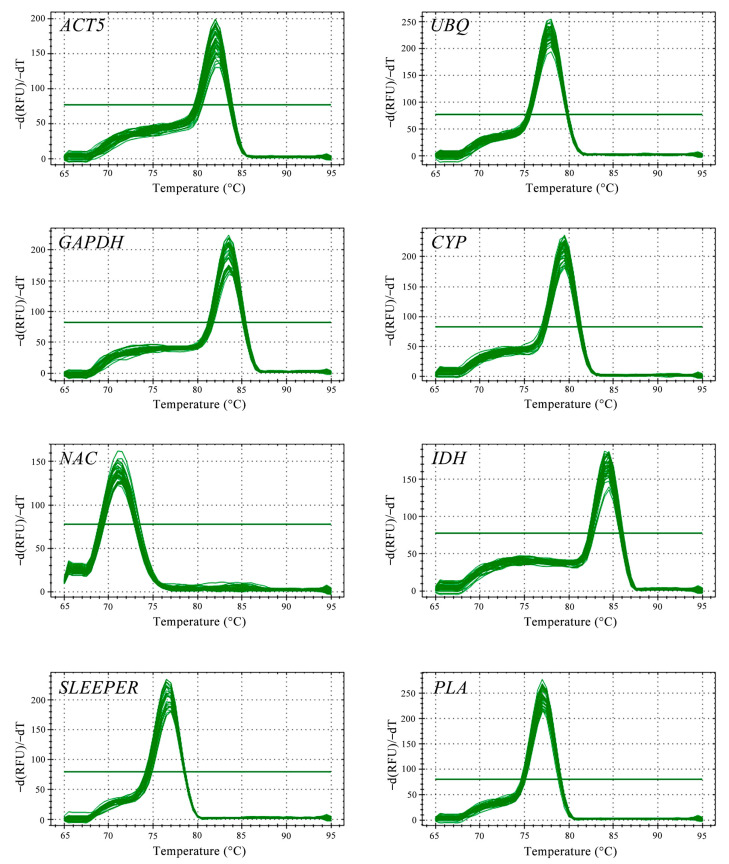
Melting curves of candidate reference genes.

PCR amplification was used to detect the specificity of primers, while electrophoresis results showed that the fragment size was correct and the band was single (Figure S3). The cDNA of all arrowhead tissues was used as the template, and melting curves of the selected reference genes were detected by qPCR. Generated melting curves showed a specific single peak, and no other miscellaneous peak was found, providing more evidence for the primer’s specificity (Figure 2). The standard curve method was employed to calculate the amplification efficiency (E) of candidate reference genes. The qPCR efficiency of eight reference genes was between 98% and 109%, and the linear correlation coefficient was above 0.99 (Table S1). The above results suggest that the specificity and amplification efficiency of the designed primers was great; the primers could be used in subsequent experiments.

### 3.2. Expression Levels of Candidate Reference Genes

**Figure 3:**
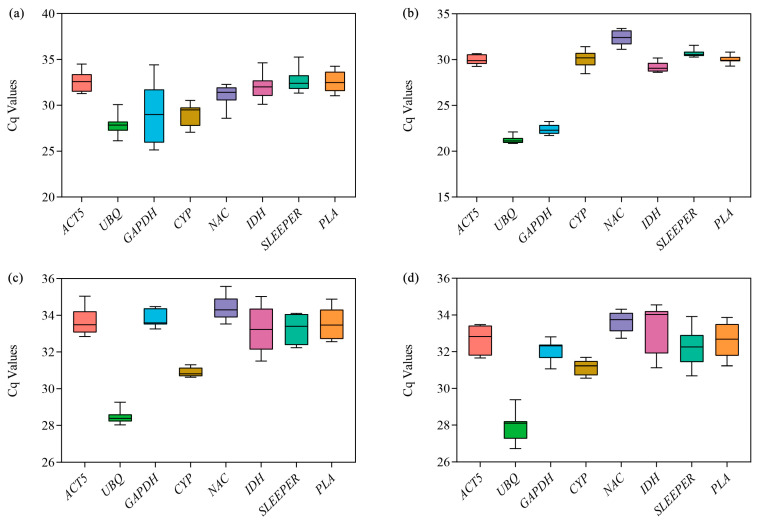
The quantification cycle (Cq) values of the candidate reference genes. (**a**) Cq of eight candidate references in different tissues. (**b**) Cq of eight candidate references in corms at different developmental stages. (**c**) Cq of eight candidate references in leaves at different developmental stages. (**d**) Cq of eight candidate references in leafstalks at different developmental stages. A line across the box-plot graph represents the median. Lower and upper boxes indicate the 25th percentile and 75th percentiles, respectively. The whisker caps represent the maximum and minimum values.

The expression levels presented as the quantification cycle (Cq) values of eight candidate reference genes in the samples were obtained by RT-qPCR assay. The Cq values of all reference genes are shown in Figure 3 and Table S2. The Cq values of reference genes in samples from different tissues and different developmental stages of the same tissue (corm, leaf and leafstalk) ranged from 25.13 (*GAPDH*) to 35.26 (*SLEEPER*), 20.85 (*UBQ*) to 33.39 (*NAC*), 28.03 (*UBQ*) to 35.57 (*NAC*) and 26.72 (*UBQ*) to 34.55 (*IDH*), respectively. Low Cq values represented high expression levels [23]. In different arrowhead tissues, the expression of *GAPDH* was the highest, while *SLEEPER* exhibited the lowest expression (Figure 3a). The expression difference of *PLA* was the smallest, whereas that of *GAPDH* was the largest. In corms of three developmental stages, the expression levels of *UBQ* and *GAPDH* were significantly higher than those of other selected genes (Figure 3b). The expression difference of *UBQ* and *CYP* was the smallest and largest, respectively. During different developmental stages of the leaf and leafstalk, the expression of *UBQ* was significantly higher than that of other genes, and the expression of *IDH* showed significant differences among the detected samples (Figure 3c,d). However, it is not accurate to identify the stably expressed reference genes simply based on the evaluation of the original Cq value. Thus, we employed the four following statistical algorithms to screen the most stable reference genes.

### 3.3. GeNorm Analysis

The GeNorm algorithm was used to calculate the M value to evaluate the expression stability of reference genes [24]. The gene with the lowest M value is considered the most stable reference gene, while the gene with the highest M value is considered to be the least stable. Regrettably, this algorithm could not distinguish the M values of the top two stable genes, resulting in two most stable reference genes in the end. The M values of each gene in materials of different groups are shown in Table 3. The GeNorm algorithm required that the M value of the appropriate reference gene be less than 1.5, indicating the expression stability of each reference gene in the samples of different groups was satisfactory. *UBQ* and *SLEEPER*, with the lowest M value (0.37), were the most stable genes expressed in different tissues, followed by *IDH* (M value of 0.40), while *GAPDH* (M value of 1.46) was the least stable gene. In corms of different developmental stages, *UBQ* and *PLA* (M value of 0.19) were the most stable genes, followed by *GAPDH* (M value of 0.24), while *CYP* (M value of 0.56) was the least stable gene. Both *SLEEPER* and *PLA* (M value of 0.32 and 0.27) were the optimum reference genes in leaf and leafstalk developmental stages.

### 3.4. NormFinder Analysis

The NormFinder algorithm was applied to evaluate the expression stability of the eight selected reference genes, the S values of all the reference genes were calculated based on intra-group and inter-group variation [25]. The lower the S values, the more stable the gene expression. The S values of each gene in the materials from different groups are listed in Table 3. In different tissues of arrowheads, the expression of *ACT5* was the most stable (S value of 0.22), followed by *UBQ* (S value of 0.25), and *GAPDH* was the least stable (S value of 2.95). In corms of different developmental stages, *IDH*, with the lowest S value (0.11), showed the highest expression stability, followed by *ACT5* (S value of 0.13), and *CYP* was the worst stable reference gene with the highest S value (0.71). *UBQ* (S value of 0.27 and 0.26) was the optimum reference gene and *IDH* (S value of 1.04 and 0.74) was the more unstable reference gene in leaves and leafstalks at different developmental stages.

### 3.5. BestKeeper Analysis

The BestKeeper algorithm was used to calculate the standard deviation (SD) and coefficient of variation (CV) to rank the expression stability of eight reference genes [26]. The smaller the SD and CV values, the more stable the gene expression. The SD and CV values of each gene in the materials from different groups are shown in Table 3. BestKeeper algorithm believes that the SD value of the appropriate reference gene should be less than 1, so *GAPDH* and *IDH* were excluded from the analysis of different tissues and different developmental stages of leaf and leafstalk in arrowheads, respectively. In different tissues, *NAC* was the most stable gene (SD value of 0.72, CV value of 2.31), followed by *UBQ* (SD value of 0.76, CV value of 2.73), and *GAPDH* was the least stable gene (SD value of 2.99, CV value of 10.24). In corms of different developmental stages, *SLEEPER* (SD value of 0.29, CV value of 0.94) and *UBQ* (SD value of 0.30, CV value of 1.40) were judged to be the top two genes in terms of stable expression, and *NAC* (SD value of 0.67, CV value of 2.06) showed the least stable expression. During the different developmental stages of leaves and leafstalks, *CYP* (SD value of 0.21 and 0.33, CV value of 0.68 and 1.05) expression was the most stable, while *IDH* (SD value of 1.00 and 1.08, CV value of 3.03 and 3.24) was, to the contrary, not stable.

### 3.6. ΔCt Analysis

The expression stability of the selected reference genes was also evaluated using the ΔCt algorithm based on the SD. The gene with the lowest S value is thought to be the most stable [27]. As shown in Table 3, the S values of each gene in the arrowhead samples from different groups were assessed. In different tissues, *UBQ* showed the lowest S value (1.04) and it was the most stable reference gene, whereas *GAPDH* (S value of 3.03) was the least stable. The expression of *IDH* was the most stable (S value of 0.44) in arrowhead corms among the three developmental stages, followed by *ACT5* (S value of 0.46), and *CYP* exhibited the least stable (S value of 0.78) expression. In leaves and leafstalks at different developmental stages, *UBQ* (S value of 0.69) and *PLA* (S value of 0.51) showed the most expression stability, while the expression of *IDH* (S value of 1.15 and 0.81) was the most unstable.

### 3.7. RefFinder Analysis

RefFinder is a comprehensive web-based tool that includes different algorithms from GeNorm, NormFinder, BestKeeper and ΔCt [22]. Based on the results of all statistic methods, the stability of the arrowhead candidate reference genes was comprehensively evaluated by RefFinder. The comprehensive analysis results are recorded in Table 3.

After comprehensive comparative analysis based on the above four algorithms, we found that *UBQ* was the most stable gene expressed in different arrowhead tissues, followed by *SLEEPER*, whereas *GAPDH* was the least stable. In different developmental stages of corms, *IDH* showed the most stable expression, followed by *UBQ*, and *CYP* obtained the lowest score in expression stability. The stability scores of *UBQ* and *PLA* were the highest during the developmental stages of leaves and leafstalks, respectively, whereas the stability score of *IDH* was the lowest.

### 3.8. Reference Gene Validation 

**Figure 4:**
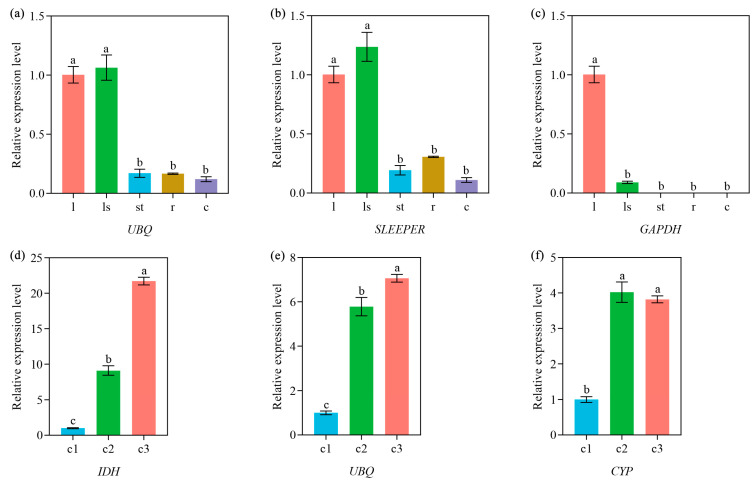
The relative expression levels of *PDS* and *EXP1* gene normalized by different reference genes. (**a**–**c**) Relative expression level of *PDS* in different arrowhead tissues. (**d**–**f**) Relative expression level of *EXP1* in corms of different developmental stages. l: leaf, ls: leafstalk, st: stolon, r: root, c: corm, c1–c3: the three stages of arrowhead corm development. Error bars represent the standard deviation of three replicates. Different lowercase letters indicate significant differences at *p*  <  0.05.

Phytoene desaturase (PDS) is a key enzyme involved in carotenoid synthesis, which also has been proved to function in regulating chlorophyll deposition in plant tissues [28]. PDS is usually highly expressed in plant tissues that appear green. *EXP1* (encoding one expansin) is the major factor regulating cell wall extension and plays an important role in plant organ expansion [29]. In order to verify the reliability and accuracy of the selected reference genes, we measured the relative expression of *PDS* in different tissues and *EXP1* in corms from different developmental stages. The results show that the relative expression levels of *PDS* in green leaf and leafstalk are significantly higher than those in three other tissues when *UBQ* and *SLEEPER* were used as the reference genes. When the most unstable reference gene *GAPDH* was used as the internal standard, *PDS* was only highly expressed in the leaf (Figure 4a–c). During the corm expansion process, stably expressed *IDH* and *UBQ* were employed as the reference gene, and the relative expression level of *EXP1* was up-regulated with the development of corms and peaked in stage 3 of corm development. When *CYP* was used as the reference gene, the *EXP1* expression pattern was overestimated at stage 2 of corm development. (Figure 4d–f). The above results demonstrate that our screening for reference genes in varied arrowhead tissues and corms of different development stages was reliable.

## 4. Discussion

In recent years, RT-qPCR has become the preferred method in detecting gene expression [30]. Normalization of data by a reference gene with a highly stable expression is a prerequisite in ensuring the reliability of experimental results [31]. In this study, the expression levels of eight candidate reference genes (*ACT5*, *UBQ*, *GAPDH*, *CYP*, *NAC*, *IDH*, *SLEEPER* and *PLA*) were determined by using the samples from different tissues and different developmental stages of the same tissue (corm, leaf, leafstalk) in arrowheads. This provided a reference for studying the physiological and biochemical processes of arrowheads at the gene expression level.

Four statistical algorithms, GeNorm, NormFinder, BestKeeper and ΔCt, were used to evaluate the stability of these selected reference genes [32]. The most stable reference genes screened from several statistical algorithms were different, while the least stable reference gene was highly consistent. In different arrowhead tissues, the combination of *UBQ* and *SLEEPER*, *ACT5*, *NAC* and *UBQ* ranked first in GeNorm, NormFinder, BestKeeper and ∆Ct algorithms, respectively, and were considered to be the most stable reference genes. Whereas *GAPDH* was judged to be the least stable reference gene throughout the analysis of four algorithms. During the expansion of corms, both Normfinder and ∆Ct algorithms considered *IDH* to be the best reference gene, Genorm and BestKeeper algorithms indicated that the combination of *UBQI, PLA* and *SLEEPER* may be the most stable, respectively. *CYP* and *NAC* constituted the least stable reference genes based on the analysis of four algorithms. In three developmental stages of leaves and leafstalks, *UBQ* was considered as the most stably expressed gene by the NormFinder algorithm and ΔCt algorithm, and the expression of *IDH* showed the least stability. Since different statistical algorithms were applied, these differences in screening of optimal internal reference genes were acceptable. Finally, the RefFinder algorithm was used to comprehensively compare and rank these reference genes [33], suggesting that *UBQ* was the best reference gene in different tissues and growth of leaves, and *IDH* and *PLA* were the optimum references for gene expression normalization during the growth of corms and leafstalks, respectively. Based on the results that mentioned above, it is concluded that *UBQ* is the most suitable reference gene in arrowhead tissues.

*UBQ* and *IDH* have also been reported as reference genes in other plants. *UBQ* was verified to be a stably expressed reference gene in different parts and seeds of different developmental stages of *Paeonia ostii* [34], while a similar report has been found in *Dimocarpus longan* [35]. IDH has been used as the reference gene under specific conditions in other species, such as different developmental stages of flowers in *Pinus massoniana*, and various stress conditions in *Eucalyptus* [36,37]. The reliable reference genes evaluated by GeNorm, NormFinder and ∆Ct in our work were similar to previous findings.

In order to verify the accuracy of the selected optimal reference genes, we detected the expression levels of *PDS* and *EXP1* in different tissues and corms of different developmental stages using different reference genes. *PDS* was closely linked to the accumulation of chlorophyll and was highly expressed in green plant tissues in general [38]. In *Triticum aestivum*, the expression of the *PDS* gene was highest in the leaf and petal, followed by the stem and inflorescence, and lowest in the decolorized root [39]. In *Andrographis paniculata*, *PDS* exhibited the highest transcription level in the leaf [38]. In *Allium sativum*, the *PDS* gene was highly expressed in green leaves compared to those in roots [40]. Considering our results in arrowheads, when *UBQ* and *SLEEPER* genes were applied to normalize gene expression, the *PDS* gene showed a significantly high expression in leaves and leafstalks, and a low expression in roots, stolons and corms. This result is consistent with the findings in previous research. However, the expression pattern of *PDS* normalized by the *GAPDH* gene presented an inconsistent result. *EXP1* encoded a class of expansins, which contributed to cell expansion during the development of plant tissue [41]. In *Psidium guajava*, the expression of *EXP1* increased with fruit ripening and reached the highest level at the ripening stage [42]. In *Solanum tuberosum*, EXP was closely related to the tuber expansion process, and the *EXP* gene exhibited high expression in growing tubers [43]. Under the normalization of *IDH* and *UBQ*, the expression level of *EXP1* during corms development was consistent with that previously described. However, the expression level of *EXP1* was overestimated at stage 2 of corm development when the unstable reference gene (*CYP*) was used to normalize gene expression. The assay of *PDS* and *EXP1* gene expression further confirmed the accuracy of the selected reference genes in arrowheads.

## 5. Conclusions

In summary, five statistical algorithms were used to compare and screen suitable reference genes in the samples from different tissues and three developmental stages of the same tissue (corm, leaf and leafstalk) in arrowheads. The results concluded that *UBQ* was the most stable reference gene in different tissues and developmental stages of leaves, while *IDH* and *PLA* were the most stable reference genes during developmental stages of corms and leafstalks, respectively. The accuracy of the selected reference genes was verified by the expression normalization of the *PDS* gene in different tissues and the *EXP1* gene in corms at three developmental stages. The current work will provide a reliable basis for the future research about the tissue- and developmental-stage-specific expression analysis of genes in arrowheads.

## Figures and Tables

**Table 1:** Candidate reference genes and primer sequences.

Gene Symbol	Gene Name	Primer Sequences (5′–3′)	
*ACT5*	*Actin 5*	Forward Reverse	CGGAGAGGCTGTCACGATT CGAACTGATAGACGACATTGGAA
*UBQ*	*Ubiquitin*	Forward Reverse	AGGTGCTACTCTCCATCTGTTC CATACTTGTTCCTGTCTGTCTTGT
*GAPDH*	*Glyceraldehyde-3-phospho* *dehydrogense*	Forward Reverse	CTCCTCCTCGCAATACTCACA CAGCCACAACTTCAACATCGT
*CYP*	*Cyclophilin*	Forward Reverse	CTCCACCTTCCACCGTATCAT GAACTTCAAGCCGTAGATAGACTC
*NAC*	*NAC domain protein*	Forward Reverse	ATTCCTCCAACTGTCTGATGAAC ACTGCCTGATATGAGCCTGTT
*IDH*	*Isocitrate dehydrogenase*	Forward Reverse	CTGAGAACAGCAGCGGTAAC GGCAACGGTCGCATAAGAG
*SLEEPER*	*Zinc finger BED domain-containing protein* *DAYSLEEPER-like*	Forward Reverse	CCTGTATTGCTTGGAAGTGGTAT CCGTCGTCTTGATTGAATGGAT
*PLA*	*Phospholipase A I*	Forward Reverse	GACCTTATCTGTGGCACTTCTAC CTCTCCAAGTTGCTGCTTCATT

**Table 2:** Primer sequences of *PDS* and *EXP1* used for RT-qPCR.

Gene Symbol	Gene Name	Primer Sequences (5′–3′)	
*PDS*	*Phytoene desaturase*	Forward Reverse	CAGTCTCCGTCTCCACCGTCAA CCACCATCCGCAAGATACTTAGCC
*EXP1*	*Expansin*	Forward Reverse	GTCAACATTGTGGTCACGGATTACG TGTGCCATCAAGCCATCAGTCAG

**Table 3:** Expression stability analysis of candidate reference genes.

Materials	Rank	GeNorm		NormFinder		BestKeeper			Delta CT		RefFinder	
		Gene	Stability	Gene	Stability	Gene	SD	CV	Gene	Stability	Gene	Values
Different tissues	1	*UBQ*	0.37	*ACT5*	0.22	*NAC*	0.72	2.31	*UBQ*	1.04	*UBQ*	1.41
	2	*SLEEPER*	0.37	*UBQ*	0.25	*UBQ*	0.76	2.73	*IDH*	1.07	*SLEEPER*	2.78
	3	*IDH*	0.40	*IDH*	0.28	*SLEEPER*	0.85	2.62	*ACT5*	1.08	*ACT5*	3.03
	4	*ACT5*	0.46	*PLA*	0.39	*PLA*	0.86	2.63	*SLEEPER*	1.10	*IDH*	3.22
	5	*PLA*	0.51	*SLEEPER*	0.51	*CYP*	0.94	3.23	*PLA*	1.12	*NAC*	3.83
	6	*NAC*	0.70	*NAC*	1.00	*IDH*	0.96	2.99	*NAC*	1.45	*PLA*	4.47
	7	*CYP*	0.94	*CYP*	1.52	*ACT5*	0.98	3.00	*CYP*	1.81	*CYP*	6.44
	8	*GAPDH*	1.46	*GAPDH*	2.95	*GAPDH*	2.99	10.24	*GAPDH*	3.03	*GAPDH*	8.00
Corm developmental stages	1	*UBQ*	0.19	*IDH*	0.11	*SLEEPER*	0.29	0.94	*IDH*	0.44	*IDH*	2.11
	2	*PLA*	0.19	*ACT5*	0.13	*UBQ*	0.30	1.40	*ACT5*	0.46	*UBQ*	2.21
	3	*GAPDH*	0.24	*SLEEPER*	0.31	*PLA*	0.32	1.05	*UBQ*	0.51	*PLA*	2.78
	4	*IDH*	0.31	*UBQ*	0.37	*GAPDH*	0.41	1.85	*PLA*	0.52	*SLEEPER*	3.08
	5	*ACT5*	0.33	*PLA*	0.40	*IDH*	0.47	1.62	*SLEEPER*	0.54	*ACT5*	3.31
	6	*SLEEPER*	0.38	*GAPDH*	0.44	*ACT5*	0.47	1.58	*GAPDH*	0.54	*GAPDH*	4.56
	7	*NAC*	0.49	*NAC*	0.64	*CYP*	0.67	2.21	*NAC*	0.73	*NAC*	7.24
	8	*CYP*	0.56	*CYP*	0.71	*NAC*	0.67	2.06	*CYP*	0.78	*CYP*	7.74
Leaf developmental stages	1	*SLEEPER*	0.32	*UBQ*	0.27	*CYP*	0.21	0.68	*UBQ*	0.69	*UBQ*	1.68
	2	*PLA*	0.32	*ACT5*	0.33	*UBQ*	0.26	0.91	*ACT5*	0.71	*ACT5*	2.78
	3	*ACT5*	0.49	*GAPDH*	0.44	*GAPDH*	0.44	1.29	*SLEEPER*	0.76	*SLEEPER*	3.03
	4	*UBQ*	0.59	*SLEEPER*	0.45	*NAC*	0.51	1.48	*GAPDH*	0.76	*CYP*	3.50
	5	*GAPDH*	0.63	*CYP*	0.52	*ACT5*	0.55	1.64	*CYP*	0.79	*GAPDH*	3.66
	6	*CYP*	0.65	*PLA*	0.57	*PLA*	0.64	1.92	*PLA*	0.81	*PLA*	3.83
	7	*NAC*	0.74	*NAC*	0.95	*SLEEPER*	0.65	1.96	*NAC*	1.06	*NAC*	6.09
	8	*IDH*	0.84	*IDH*	1.04	*IDH*	1.00	3.03	*IDH*	1.15	*IDH*	8.00
Leafstalk developmental stages	1	*SLEEPER*	0.27	*UBQ*	0.26	*CYP*	0.33	1.05	*PLA*	0.51	*PLA*	1.93
	2	*PLA*	0.27	*PLA*	0.27	*GAPDH*	0.42	1.33	*UBQ*	0.51	*UBQ*	2.21
	3	*UBQ*	0.35	*ACT5*	0.33	*NAC*	0.45	1.33	*SLEEPER*	0.54	*SLEEPER*	2.91
	4	*ACT5*	0.43	*SLEEPER*	0.34	*UBQ*	0.56	2.01	*ACT5*	0.54	*CYP*	3.50
	5	*GAPDH*	0.47	*CYP*	0.41	*ACT5*	0.65	1.97	*CYP*	0.56	*ACT5*	3.94
	6	*CYP*	0.48	*GAPDH*	0.44	*SLEEPER*	0.74	2.31	*GAPDH*	0.58	*GAPDH*	4.36
	7	*NAC*	0.51	*NAC*	0.47	*PLA*	0.75	2.30	*NAC*	0.62	*NAC*	5.66
	8	*IDH*	0.59	*IDH*	0.74	*IDH*	1.08	3.24	*IDH*	0.81	*IDH*	8.00

## Data Availability

The data used to support the findings of this study are included within the article.

## Supplementary Materials

The following supporting information can be downloaded at https://www.mdpi.com/article/10.3390/genes14071321/s1. Figure S1: The ORF sequence of *PDS* gene in ‘BMT’ arrowhead. Figure S2: The ORF sequence of *EXP1* gene in ‘BMT’ arrowhead. Figure S3: PCR amplification of specific primers for selected candidate reference genes. Table S1: Amplification efficiency of candidate reference genes primer pairs. Table S2: The quantification cycle (Cq) values of the candidate reference genes.

## References

[1] Brattelid T., Levy F.O. (2011). Quantification of GPCR mRNA using real-time RT-PCR. Methods Mol. Biol..

[2] Niu L., Tao Y., Chen M., Fu Q., Li C., Dong Y., Wang X., He H., Xu Z. (2015). Selection of reliable reference genes for gene expression studies of a promising oilseed crop, *Plukenetia volubilis*, by real-time quantitative PCR. Int. J. Mol. Sci..

[3] Sun Z., Li S., Sun M. (2015). Selection of reliable reference genes for gene expression studies in *Clonostachys rosea* 67-1 under sclerotial induction. J. Microbiol. Methods.

[4] Gao D., Kong F., Sun P., Bi G., Mao Y. (2018). Transcriptome-wide identification of optimal reference genes for expression analysis of *Pyropia yezoensis* responses to abiotic stress. BMC Genomics.

[5] Sinha P., Singh V.K., Suryanarayana V., Krishnamurthy L., Saxena R.K., Varshney R.K. (2015). Evaluation and validation of housekeeping genes as reference for gene expression studies in pigeonpea (*Cajanus cajan*) under drought stress conditions. PLoS ONE.

[6] Dheda K., Huggett J.F., Chang J.S., Kim L.U., Bustin S.A., Johnson M.A., Rook G.A.W., Zumla A. (2005). The implications of using an inappropriate reference gene for real-time reverse transcription PCR data normalization. Anal. Biochem..

[7] Wang M., Wang Q., Zhang B. (2013). Evaluation and selection of reliable reference genes for gene expression under abiotic stress in cotton (*Gossypium hirsutum* L.). Gene.

[8] Andersen C.L., Jensen J.L., Orntoft T.F. (2004). Normalization of real-time quantitative reverse transcription-PCR data: A model-based variance estimation approach to identify genes suited for normalization, applied to bladder and colon cancer data sets. Cancer Res..

[9] Zhu X., Wang B., Wang X., Wei X. (2021). Screening of stable internal reference gene of Quinoa under hormone treatment and abiotic stress. Physiol. Mol. Biol. Plants.

[10] Popovici V., Goldstein D.R., Antonov J., Jaggi R., Delorenzi M., Wirapati P. (2009). Selecting control genes for RT-QPCR using public microarray data. BMC. Bioinformatics.

[11] Wang G., Tian C., Wang Y., Wan F., Hu L., Xiong A., Tian J. (2019). Selection of reliable reference genes for quantitative RT-PCR in garlic under salt stress. PeerJ.

[12] Dheda K., Huggett J.F., Bustin S.A., Johnson M.A., Rook G., Zumla A. (2004). Validation of housekeeping genes for normalizing RNA expression in real-time PCR. Biotechniques.

[13] Gambarotta G., Ronchi G., Friard O., Galletta P., Perroteau I., Geuna S. (2014). Identification and validation of suitable housekeeping genes for normalizing quantitative real-time PCR assays in injured peripheral nerves. PLoS ONE.

[14] Itoh K. (1997). Ecological studies on tubers of arrowhead (*Sagittaria trifolia* L.), a perennial weed in the paddy fields. Bull. Natl. Agric. Res. Cent..

[15] Zhang Y., Yang G., Wang X., Ni G., Cui Z., Yan Z. (2021). *Sagittaria trifolia* tuber: Bioconstituents, processing, products, and health benefits. J. Sci. Food Agric..

[16] Devi R., Kumar S. (2019). *Sagittaria trifolia* L.: A potential nutraceutical of the Northeastern part of India. Biodivers. Conserv..

[17] Vandesompele J., De Preter K., Pattyn F., Poppe B., Van Roy N., De Paepe A., Speleman F. (2002). Accurate normalization of real-time quantitative RT-PCR data by geometric averaging of multiple internal control genes. Genome Biol..

[18] Andersen C.L., Jensen J.L., Ørntoft T.F. (2004). Normalization of real-time quantitative reverse transcription-PCR data: A mod-el-based variance estimation approach to identify genes suited for normalization, applied to bladder and colon cancer data sets. Cancer Res..

[19] Lin L., Han X., Chen Y., Wu Q., Wang Y. (2013). Identification of appropriate reference genes for normalizing transcript expression by quantitative real-time PCR in Litsea cubeba. Mol. Genet. Genomics.

[20] Pfaffl M.W., Tichopad A., Prgomet C., Neuvians T.P. (2004). Determination of stable housekeeping genes, differentially regulated target genes and sample integrity: BestKeeper—Excel-based tool using pair-wise correlations. Biotechnol. Lett..

[21] Silver N., Best S., Jiang J., Thein S.L. (2006). Selection of housekeeping genes for gene expression studies in human reticulocytes using real-time PCR. BMC Mol. Biol..

[22] Xie F., Wang J., Zhang B. (2023). RefFinder: A web-based tool for comprehensively analyzing and identifying reference genes. Funct. Integr. Genomics.

[23] Yang Z., Zhang R., Zhou Z. (2021). Identification and validation of reference genes for gene expression analysis in *Schima superba*. Genes.

[24] Gantasala N.P., Papolu P.K., Thakur P.K., Kamaraju D., Sreevathsa R., Rao U. (2013). Selection and validation of reference genes for quantitative gene expression studies by real-time PCR in eggplant (*Solanum melongena* L.). BMC Res. Notes.

[25] Wang M., Bhullar N.K. (2021). Selection of suitable reference genes for qRT-PCR gene expression studies in rice. Methods Mol. Biol..

[26] Brunner A.M., Yakovlev I.A., Strauss S.H. (2004). Validating internal controls for quantitative plant gene expression studies. BMC Plant Biol..

[27] Aggarwal A., Jamwal M., Viswanathan G.K., Sharma P., Sachdeva M.S., Bansal D., Malhotra P., Das R. (2018). Optimal reference gene selection for expression studies in human reticulocytes. J. Mol. Diagn..

[28] Zhu Y., Jiang J., Yan Y., Chen X. (2005). Isolation and characterization of phytoene desaturase cDNA involved in the beta-carotene biosynthetic pathway in Dunaliella salina. J. Agric. Food Chem..

[29] Wu Y., Meeley R.B., Cosgrove D.J. (2001). Analysis and expression of the alpha-expansin and beta-expansin gene families in maize. Plant Physiol..

[30] Li J., Han X., Wang C., Qi W., Zhang W., Tang L., Zhao X. (2017). Validation of suitable reference genes for RT-qPCR data in *Achyranthes bidentata* Blume under different experimental conditions. Front. Plant Sci..

[31] Bu J., Zhao J., Liu M. (2016). Expression stabilities of candidate reference genes for RT-qPCR in Chinese jujube (*Ziziphus jujuba* Mill.) under a variety of conditions. PLoS ONE.

[32] Yuan Z., Zhang X., Pang Y., Qi Y., Wang Q., Ren S., Hu Y., Zhao Y., Wang T., Huo L. (2022). Screening of stably expressed internal reference genes for quantitative real-time PCR analysis in quail. Biol. Bull..

[33] Chen R., Chen W., Tigabu M., Zhong W., Li Y., Ma X., Li M. (2019). Screening and evaluation of stable reference genes for quantitative real-time polymerase chain reaction (qRT-PCR) analysis in Chinese fir roots under water, phosphorus, and nitrogen stresses. Forests.

[34] Li C., Hu L., Wang X., Liu H., Tian H., Wang J. (2019). Selection of reliable reference genes for gene expression analysis in seeds at different developmental stages and across various tissues in *Paeonia ostii*. Mol. Biol. Rep..

[35] Lin Y., Lai Z. (2010). Reference gene selection for qPCR analysis during somatic embryogenesis in longan tree. Plant Sci..

[36] Moura J.C.M.S., Araujo P., Brito M.d.S., Souza U.R., Viana J.d.O.F., Mazzafera P. (2012). Validation of reference genes from *Eucalyptus* spp. under different stress conditions. BMC Res. Notes.

[37] Chen H., Yang Z., Hu Y., Tan J., Jia J., Xu H., Chen X. (2016). Reference genes selection for quantitative gene expression studies in *Pinus massoniana* L.. Trees-Struct. Funct..

[38] Choi S., Seo Y.B., Han Kyu L., Soo-Wan N., Gun-Do K. (2018). Molecular cloning and overexpression of phytoene desaturase (CrtI) from *Paracoccus haeundaensis*. Microbiol. Biotechnol. Lett..

[39] Cong L., Wang C., Li Z., Chen L., Yang G., Wang Y., He G. (2010). cDNA cloning and expression analysis of wheat (*Triticum aestivum* L.) phytoene and zeta-carotene desaturase genes. Mol. Biol. Rep..

[40] Tuan P.A., Kim J.K., Kim H.H., Lee S.Y., Park N.I., Park S.U. (2011). Carotenoid accumulation and characterization of cDNAs encoding phytoene synthase and phytoene desaturase in Garlic (*Allium sativum*). J. Agric. Food Chem..

[41] Zhou Y., Luo S., Hameed S., Xiao D., Zhan J., Wang A., He L. (2020). Integrated mRNA and miRNA transcriptome analysis reveals a regulatory network for tuber expansion in Chinese yam (*Dioscorea opposita*). BMC Genomics.

[42] Silva A.I.R., Palenius H.G.N., Guzman G.H., Solis A.G.A., Pina C.G., Dominguez J.F.M. (2013). Ripening-related cDNAs in guava fruit (*Psidium guajava* L.). Characterization and expression analysis. Rev. Fitotec. Mex..

[43] Mao Y. (2013). Research progress of tomato and potato extension protein. J. Heilongjiang Bayi Agric. Univ..

