# Supplementary material for: Selection of Reliable Reference Genes for Gene Expression Normalization in Sagittaria trifolia
Source: Genes (Basel). 2023 Jun 23;14(7):1321. doi: 10.3390/genes14071321 (PMC10379039; doi:10.3390/genes14071321)
Supplement: Supplementary file 1 [file genes-14-01321-s001.zip › genes-2393969-supplementary.pdf]

Selection of reliable reference genes for gene expression normalization in *Sagittaria trifolia*

**Jing Tang[1], Enjiao Li[1], Jiexia Liu[1], Zhiping Zhang[1], Bing Hua[1], Jiezeng Jiang[1], Minmin Miao[1,2,3]*

[1]College of Horticulture and Landscape, Yangzhou University, Yangzhou 225009, China
[2]Joint International Research Laboratory of Agriculture and Agri-Product Safety of Ministry of Education of China, Yangzhou University, Yangzhou 225009, China
[3]Key Laboratory of Plant Functional Genomics of the Ministry of Education/Jiangsu Key Laboratory of Crop Genomics and Molecular Breeding, Yangzhou University, Yangzhou 225009, China

***Corresponding author:** Minmin Miao
Telephone: +86-0514-87979344;
Fax: +86-0514-87347537;
Email: mmmiao@yzu.edu.cn.

The author responsible for distribution of materials integral to the findings presented in this article in accordance with the policy described in the Instructions for Authors (https://academic.oup.com/plcell/pages/General-Instructions) is: Minmin Miao (mmmiao@yzu.edu.cn).

Supplementary data

```
1       ATGGCGGGAGCAGTGAAGAGTATCCTCGCAGTCTCCGTCTCCACCGTCAATTTCGGTATCACCGATTCTGGAGACCGGAGAAGCAGCAAT
        M  A  G  A  V  K  S  I  L  A  V  S  V  S  T  V  N  F  G  I  T  D  S  G  D  R  R  S  S  N
91      TCGCTCGGGTACATAAACAGCGATGCCTTCGGCTGCCGCCTGAAAGCACCGAGCTTGTCGCTCGACGGAGCCGGGCGTAGGCGGGGAACA
        S  L  G  Y  I  N  S  D  A  F  G  C  R  L  K  A  P  S  L  S  L  D  G  A  G  R  R  R  G  T
181     TCCCGTTTGCAGATTGTCTGCAGGGATTTTCCCAGGCCTGAGCTGGAAAACACTGCTAATTTTTAGAAGCTGCATCAATGTCTTCATCC
        S  R  L  Q  I  V  C  R  D  F  P  R  P  E  L  E  N  T  A  N  F  L  E  A  A  S  M  S  S  S
271     TTCAGAAGTTCTCCTCGACCTAGTAAGCCCTTGAAAGTGGTGATTGCTGGCGCAGGTCTGGCTGGTCTATCAACGGCTAAGTATCTTGCG
        F  R  S  S  P  R  P  S  K  P  L  K  V  V  I  A  G  A  G  L  A  G  L  S  T  A  K  Y  L  A
361     GATGGTGGGCATAAGCCTATTCTCTTGGAAGCTAGAGATGTTTTAGGTGGCAAGATAGCGGCTTGGAAGGATGATGACGGGGACTGGTAT
        D  G  G  H  K  P  I  L  L  E  A  R  D  V  L  G  G  K  I  A  A  W  K  D  D  D  G  D  W  Y
451     GAGACGGGCCTGCATATATTTTTTGGAGCATATCCGAATGTTCAAAATCTCTTTGGTGAACTTGGAATTAATGATCGTTTACAATGGAAG
        E  T  G  L  H  I  F  F  G  A  Y  P  N  V  Q  N  L  F  G  E  L  G  I  N  D  R  L  Q  W  K
541     GAACACTCGATGATATTTGCGATGCCGAACAAACCAGGAGAATTCAGCCGGTTCGATTTCCCTGAAGTTCTTCCTGCTCCATTGAATGGA
        E  H  S  M  I  F  A  M  P  N  K  P  G  E  F  S  R  F  D  F  P  E  V  L  P  A  P  L  N  G
631     ATATGGGCTATCTTAAGAAATAATGAAATGCTGACTTGGCCGGAGAAAGTAAAATTTGCTATTGGGTTGTTACCTGCAATGCTTGGTGGC
        I  W  A  I  L  R  N  N  E  M  L  T  W  P  E  K  V  K  F  A  I  G  L  L  P  A  M  L  G  G
721     CAGTCTTATGTTGAAGCTCAGGATAGTTTAACAGTAAAGGAATGGATGAAAAAACAGGGAGTACCTGATCGGGTCAATGATGAAGTTTTC
        Q  S  Y  V  E  A  Q  D  S  L  T  V  K  E  W  M  K  K  Q  G  V  P  D  R  V  N  D  E  V  F
811     ATTGCAATGTCGAAAGCTCTTAATTTTATAAATCCTGATGAGCTTTCCATGCAATGCATCTTGATTGCCCTGAACCGTTTCCTACAGGAG
        I  A  M  S  K  A  L  N  F  I  N  P  D  E  L  S  M  Q  C  I  L  I  A  L  N  R  F  L  Q  E
901     AAACATGGGTCAAAGATGGCATTTTTAGATGGCAATCCACCAGAGAGATTGTGCCTACCAATTGTTAATCATATTGAGTCGTTGGGTGGT
        K  H  G  S  K  M  A  F  L  D  G  N  P  P  E  R  L  C  L  P  I  V  N  H  I  E  S  L  G  G
991     GAGGTTCGACTCAATTCACGTATTCAAAAGATTGAACTCAATTCTGACAATACTGTCAAGCATTTTGTGCTCAATAATGGAAGTGTGATT
        E  V  R  L  N  S  R  I  Q  K  I  E  L  N  S  D  N  T  V  K  H  F  V  L  N  N  G  S  V  I
1081    GACGGAGATGCTTATGTGGTTGCTACTCCAGTTGACATATTCAAGCTCCTTTTGCCTCATGAATGGAAAGAAATTCAATACTTTAAACGG
        D  G  D  A  Y  V  V  A  T  P  V  D  I  F  K  L  L  L  P  H  E  W  K  E  I  Q  Y  F  K  R
1171    TTGGAGAAATTAGTTGGTGTCCCCGTGATTAACGTTCATATCTGGTTTGACAGGAAGCTGAAGAACACATATGACCATCTTCTTTTTAGC
        L  E  K  L  V  G  V  P  V  I  N  V  H  I  W  F  D  R  K  L  K  N  T  Y  D  H  L  L  F  S
1261    AGGAGTCCTCTTTTAAGTGTATATGCAGACATGTCTCTAGCATGCAAGGAATACTACAATCCAAATCAGTCCATGCTTGAGCTTGTTTTC
        R  S  P  L  L  S  V  Y  A  D  M  S  L  A  C  K  E  Y  Y  N  P  N  Q  S  M  L  E  L  V  F
1351    GCACCCGCAGAAAAGTGGATATCATGCAGTGACAATGAAATCATTGATGCTACTATGGCAGAGCTAGCTAAACTCTTCCCCGATGAGATT
        A  P  A  E  K  W  I  S  C  S  D  N  E  I  I  D  A  T  M  A  E  L  A  K  L  F  P  D  E  I
1441    TCTGTAGATCAAAGCAAAGCCAAAATTGTGAAATATAAAGTAGTAAAAACACCGAGATCTGTTTACAAGACCATTCCTGATTGTGAACCC
        S  V  D  Q  S  K  A  K  I  V  K  Y  K  V  V  K  T  P  R  S  V  Y  K  T  I  P  D  C  E  P
1531    TGTCGCCCACTGCAAAGATCTCCTGTTGAAGGCTTTTACTTGTCTGGTGACTACACCAAACAAAAATACCTGGCTTCCATGGAGGGGGCT
        C  R  P  L  Q  R  S  P  V  E  G  F  Y  L  S  G  D  Y  T  K  Q  K  Y  L  A  S  M  E  G  A
1621    GTTTTATCTGGGAAGCTCTGTGCACAGTCTATTGTACAGGACTATGACTTGCTAGTGGCCAGAGCAGCTAGCAGACCGCGAAGTGAGCTG
        V  L  S  G  K  L  C  A  Q  S  I  V  Q  D  Y  D  L  L  V  A  R  A  A  S  R  P  R  S  E  L
1711    ACAGTTGCATGA
        T  V  A  *
```

Figure S1 The ORF sequence of *PDS* gene in 'BMT' arrowhead.

1 ATGGCTATGCCGCTCGCATTTCCTACCTTCCTGACTCTGCTCATTTTTTCCTCTCTGCTCTCCTCAGGCCAGAGCACGTTCACCTGCTCC

M A M P L A F P T F L T L L I F S S L L S S G Q S T F T C S

91 AAGGCCGTCTACTATGGCAGCCCTCTCTGTCCAGCAACCCCTAAGGGAGCCTGCGGGTACGGGGAGCTCGGCAGACAAATGAACAGCGGC

K A V Y Y G S P L C P A T P K G A C G Y G E L G R Q M N S G

181 TATGTTGCTGCTGTCTCCAGGTTATTCAGGCACGGCACTGGATGTGGTGCTTGCTATCAGATCCGGTGCAAGTATCCCCAGCTCTGTGAC

Y V A A V S R L F R H G T G C G A C Y Q I R C K Y P Q L C D

271 GACAATGGCGTCAACATTGTGGTCACGGATTACGGCTCCAGTGATGGTGTGGACTTCATATTGAGCGGTGCCGCTTTTGCGAAGCTAGCG

D N G V N I V V T D Y G S S D G V D F I L S G A A F A K L A

361 AAGTCTGGCCTGACTGATGGCTTGATGGCACATGGCTATGCTGATATCGAGTACCGCAGGATTCCATGCCAATACCAAGGATACAATCTC

K S G L T D G L M A H G Y A D I E Y R R I P C Q Y Q G Y N L

451 ATGCTCAAGATCACTGAACACAGCACATACCCCCATTACATAGCTCTTCTTCCTCTGTACCAAGGGGGGCAGAAAGACATCACGGCCATG

M L K I T E H S T Y P H Y I A L L P L Y Q G G Q K D I T A M

541 GAAATCTGGCAGGAGGATTGTCAGCAGTGGAAACCAATGAGAAAACCTTATGGCGCAGTATGGGATAAAGAGAACCCTCCTAGGGGTCCT

E I W Q E D C Q Q W K P M R K P Y G A V W D K E N P P R G P

631 TTAAACTTGAGGTTCCTGTCATGTGGAGATGATGGACAGAATTGGGTGCAGCTCAACGCTGTTATTCCTTACGATTGGAAGCCAGGAGCT

L N L R F L S C G D D G Q N W V Q L N A V I P Y D W K P G A

721 GTTTATGACTCAAACACGCAGCTCTCTTGA

V Y D S N T Q L S *

Figure S2 The ORF sequence of *EXP1* gene in ‘BMT’ arrowhead.

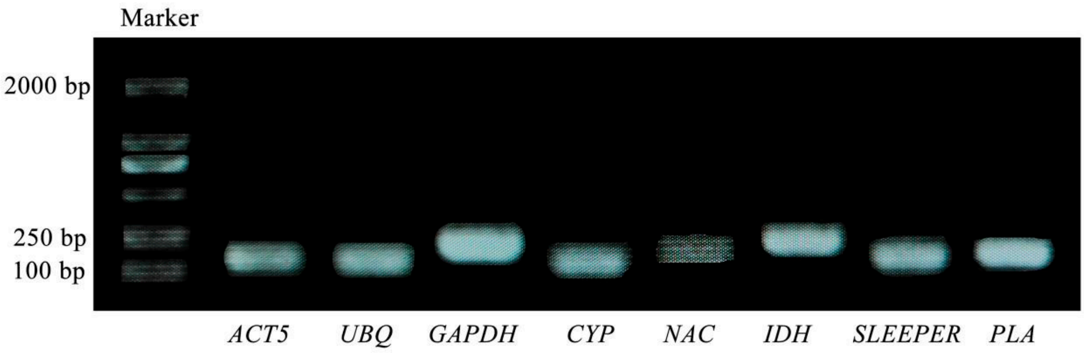

Figure S3 PCR amplification of specific primers for selected candidate reference genes.

Table S1 Amplification efficiency of candidate reference genes primer pairs.

Primer name	Slope	R^2	Amplification efficiency
ACT5	-3.3755	0.999	98%
UBQ	-3.3607	0.9959	98%
GAPDH	-3.1398	0.9977	108%
CYP	-3.1454	0.9979	108%
NAC	-3.2381	0.9976	104%
IDH	-3.1944	0.9995	106%
SLEEPER	-3.1154	0.9973	109%
PLA	-3.2283	0.9991	104%

Table S2 The quantification cycle (Cq) values of the candidate reference genes.

	ACT5	*UBQ*	*GAPDH*	*CYP*	*NAC*	*IDH*	*SLEEPER*	*PLA*
	33.10	28.18	34.44	29.31	32.28	32.18	33.18	33.68
leaf-1	33.67	28.44	34.01	29.30	31.87	32.59	33.40	33.81
	33.32	28.07	34.16	29.19	31.90	32.74	33.56	33.75
	34.33	28.81	34.29	30.00	30.35	33.80	33.32	33.57
leaf-2	34.35	28.64	34.13	30.10	30.34	34.18	33.37	33.93
	34.52	28.67	34.38	29.77	30.43	33.64	33.13	33.51
	32.90	27.87	34.05	27.82	31.57	32.07	32.58	33.56
leaf-3	32.59	27.97	34.48	27.68	31.67	32.07	32.32	33.34
	33.10	27.69	34.66	27.74	31.70	32.50	32.72	33.70
	32.92	28.14	30.96	30.76	32.39	31.94	32.27	32.42
leafstalk-1	32.64	28.09	30.95	30.53	32.18	31.89	32.30	32.63
	33.01	27.78	31.37	30.30	32.26	32.18	32.60	32.48
	32.64	28.01	31.66	29.56	31.22	32.14	32.61	32.58
leafstalk-2	32.40	28.02	31.86	29.50	31.37	32.31	32.23	32.32
	32.69	28.15	31.70	29.75	30.83	32.41	32.55	32.55
	31.76	27.43	30.62	29.77	31.35	31.40	31.95	31.69
leafstalk-3	31.72	27.27	30.11	29.72	31.47	31.11	32.48	31.43
	32.13	27.43	30.51	29.79	31.43	31.26	32.44	31.54
	34.61	30.04	30.92	29.27	32.30	34.87	34.94	33.57
root-1	34.28	30.12	30.94	29.20	31.79	34.31	35.34	33.88
	34.61	30.06	30.89	29.28	31.76	34.70	35.51	33.65
	33.19	28.01	28.60	29.34	31.90	32.61	33.00	33.23
root-2	33.68	28.04	28.11	29.82	31.90	32.75	33.26	32.67
	33.31	28.05	28.34	29.60	32.11	32.80	33.28	33.22
	33.25	28.59	28.86	27.27	31.18	33.34	34.44	34.55
root-3	33.67	28.89	28.93	26.72	31.22	32.78	34.17	34.19
	33.51	28.64	29.19	27.21	31.41	33.23	34.32	34.03
	31.38	27.17	25.10	29.65	30.52	31.01	32.06	32.06
corm-1	31.16	27.21	25.17	29.67	30.47	31.52	31.79	32.43
	31.37	27.29	25.13	30.00	30.60	31.22	31.51	32.34
	31.10	27.48	25.29	27.05	31.30	30.87	31.98	32.28
corm-2	31.37	27.53	25.16	27.19	31.61	31.09	31.88	31.83
	31.34	27.44	25.29	27.00	31.32	31.13	31.85	31.80
	31.24	27.54	25.42	30.33	30.59	32.20	32.33	32.37
corm-3	31.46	27.65	25.33	30.29	30.94	31.92	32.11	32.24
	31.73	27.54	25.34	30.42	30.71	31.65	32.05	32.17
	31.44	25.99	26.02	28.57	28.53	29.89	31.37	30.73
stolon-1	31.00	26.21	25.85	29.02	28.70	30.40	31.04	31.16
	31.44	26.18	25.92	29.09	28.52	30.05	31.55	31.22

	31.31	26.35	26.20	27.46	30.22	30.77	31.34	31.41
stolon-2	31.61	26.21	26.22	27.40	30.19	30.80	31.30	31.30
	31.78	26.28	26.30	27.48	30.23	30.37	31.61	31.24
	31.49	26.33	26.12	29.35	32.09	30.67	31.36	31.31
stolon-3	31.65	26.62	26.07	29.51	31.97	31.08	31.07	31.22
	31.39	26.48	26.11	29.65	31.70	30.63	31.56	31.74
	29.13	21.18	22.27	29.02	31.12	28.45	30.57	29.66
corm1-1	29.29	21.13	22.33	28.99	31.00	28.68	30.59	29.99
	29.38	21.13	22.14	28.74	31.22	28.73	30.38	29.84
	29.38	21.06	22.65	28.55	31.00	28.93	30.70	30.21
corm1-2	29.83	21.11	22.52	28.39	31.13	29.05	30.33	30.08
	29.56	21.20	22.53	28.46	31.47	29.21	30.60	30.28
	30.49	22.11	23.21	30.05	32.40	29.61	30.37	30.64
corm1-3	30.59	22.16	23.29	29.76	32.18	29.78	30.43	31.11
	30.50	22.05	23.27	29.75	32.13	29.67	30.55	30.74
	29.34	20.89	22.11	30.15	32.18	28.72	30.17	29.21
corm2-1	29.71	20.91	21.92	30.27	32.21	28.77	30.53	29.44
	29.56	20.74	21.90	30.14	32.07	28.74	30.14	29.21
	30.28	21.12	22.42	31.04	33.09	29.73	31.05	29.93
corm2-2	30.22	21.02	22.39	30.92	32.98	29.52	31.11	29.86
	30.24	21.09	22.45	30.67	32.91	29.58	31.11	30.00
	30.78	21.67	23.19	31.37	33.30	30.20	31.93	30.52
corm2-3	30.41	21.73	23.19	31.26	33.31	30.04	31.61	30.50
	30.77	21.64	23.09	31.64	33.55	30.28	31.16	30.22
	29.75	20.97	21.82	30.44	32.40	28.97	30.30	29.78
corm3-1	29.78	20.83	21.85	30.26	32.50	28.90	30.15	30.09
	30.15	20.89	21.79	30.01	32.32	29.01	30.55	29.79
	30.75	21.17	22.30	30.43	33.42	29.41	30.49	30.12
corm3-2	30.78	21.22	22.24	30.92	33.17	29.88	30.80	30.02
	30.44	21.25	22.34	30.50	33.58	29.48	30.63	30.21
	29.54	20.84	21.73	29.96	32.80	28.50	30.78	29.82
corm3-3	29.50	20.97	21.67	30.23	32.82	28.81	30.79	29.54
	29.51	20.94	21.77	30.16	33.20	28.64	30.44	30.08
	33.44	28.22	33.44	31.67	34.60	32.29	33.93	34.30
leaf1-1	33.44	27.89	33.06	31.15	34.32	32.00	34.24	34.79
	33.45	27.97	33.25	31.07	34.66	32.08	34.12	34.88
	34.31	28.66	33.21	31.46	33.69	34.93	33.97	34.09
leaf1-2	34.13	28.56	33.86	31.18	33.53	34.94	34.28	33.88
	34.06	28.52	33.35	31.02	34.08	35.18	34.01	33.91
	35.23	29.19	34.30	31.04	33.86	35.08	34.14	34.91
leaf1-3	35.01	29.29	34.32	30.54	33.47	34.75	33.94	34.91
	34.89	29.30	34.56	30.58	33.23	35.09	34.04	34.82
leaf2-1	33.40	28.10	34.39	30.60	35.42	32.66	33.31	33.57
	33.79	28.51	34.28	30.79	35.72	32.32	33.40	33.48

	33.56	28.43	34.41	30.51	35.59	32.48	33.49	33.67
	33.60	28.30	34.23	30.99	35.40	33.50	33.26	33.46
leaf2-2	33.32	28.52	34.16	30.53	35.46	33.38	33.95	33.33
	33.51	28.96	34.31	30.75	35.08	33.61	33.62	33.59
	34.21	28.23	34.23	30.83	34.33	33.76	33.25	33.38
leaf2-3	34.28	28.50	34.71	30.78	34.13	33.72	33.09	33.29
	34.28	28.41	34.48	30.81	34.08	33.80	33.04	33.53
	32.98	28.25	33.56	31.19	34.40	31.44	32.52	32.66
leaf3-1	32.63	28.32	33.69	30.87	34.67	31.51	32.33	32.50
	32.90	28.35	33.46	31.21	34.49	31.55	32.44	32.94
	33.04	28.45	33.43	31.05	34.37	33.21	32.16	32.27
leaf3-2	33.35	28.69	33.47	30.68	34.24	33.32	32.20	32.79
	33.25	28.70	33.85	31.10	34.25	33.16	32.57	32.61
	33.16	28.09	33.42	30.54	33.93	31.95	32.05	32.53
leaf3-3	32.85	28.14	33.61	30.55	33.68	32.37	32.47	32.86
	32.73	28.13	33.56	30.80	34.33	32.10	32.18	32.73
	33.52	28.11	32.30	31.34	33.54	34.16	32.68	34.15
leafstalk1-1	33.55	28.14	32.28	31.46	33.99	34.16	32.94	33.31
	33.33	28.05	32.36	31.22	33.60	33.77	33.01	33.20
	33.18	28.19	32.04	31.81	33.96	34.87	32.69	33.41
leafstalk1-2	33.24	28.22	31.81	31.67	34.56	34.11	33.00	33.43
	33.12	28.19	31.93	31.59	34.42	34.66	33.10	33.55
	33.41	29.18	32.56	31.44	34.39	34.22	33.95	33.98
leafstalk1-3	33.31	29.49	32.98	31.78	33.86	34.31	33.80	33.86
	33.40	29.48	32.86	31.73	33.95	34.50	33.97	33.75
	33.04	28.19	32.67	31.53	34.36	33.79	32.69	33.23
leafstalk2-1	32.80	28.28	32.22	31.31	34.31	34.29	32.39	33.45
	32.62	28.19	32.12	30.97	33.85	34.17	32.13	33.12
	32.09	28.06	31.87	30.49	33.66	33.46	31.79	32.39
leafstalk2-2	31.72	28.03	31.47	30.47	33.75	33.20	31.41	32.03
	31.60	27.90	31.57	31.10	33.80	33.54	31.89	32.03
	33.67	28.14	32.08	31.11	33.99	34.05	32.26	32.78
leafstalk2-3	33.29	28.17	32.66	31.36	34.07	34.12	32.26	32.63
	33.45	28.10	32.22	31.19	33.67	33.91	32.22	32.61
	31.71	26.97	31.72	30.72	32.65	31.41	31.34	31.41
leafstalk3-1	31.70	26.92	31.68	30.74	33.02	30.99	30.79	31.22
	31.88	26.91	31.60	30.78	32.53	31.15	31.34	31.55
	31.88	26.70	31.00	30.57	33.52	31.04	30.52	31.17
leafstalk3-2	31.64	26.70	31.04	30.63	33.16	31.04	30.79	31.30
	31.43	26.74	31.17	30.49	33.14	31.31	30.76	31.22
	32.46	27.47	32.62	31.38	32.98	32.45	32.04	32.50
leafstalk3-3	32.89	27.59	32.41	31.19	32.78	32.67	32.03	32.74
	32.49	27.66	32.24	31.13	33.06	32.72	31.73	32.60
